# Association between atherogenic index of plasma control level and incident cardiovascular disease in middle-aged and elderly Chinese individuals with abnormal glucose metabolism

**DOI:** 10.1186/s12933-024-02144-y

**Published:** 2024-02-08

**Authors:** Qianqian Min, Zhigang Wu, Jiangnan Yao, Siyi Wang, Lanzhi Duan, Sijia Liu, Mei Zhang, Yanhong Luo, Dongmei Ye, Yuxu Huang, Lan Chen, Ke Xu, Jianghua Zhou

**Affiliations:** 1 Department of Cardiology, The First Affiliated Hospital of Wenzhou Medical University, Wenzhou, China; 2 Department of Neurology, Affiliated Hospital of Jinggangshan University, Jinggangshan University, Ji’an, Jiangxi province China; 3 Department of Endocrinology, The First Affiliated Hospital of Wenzhou Medical University, Wenzhou, China; 4 Department of medicine, Jinggangshan University, Ji’an, Jiangxi province China; 5 College of Nursing, Wenzhou Medical University, Wenzhou, China; 6 Key Laboratory of Diagnosis and Treatment of Severe Hepato-Pancreatic Diseases of Zhejiang Province, The First Affiliated Hospital of Wenzhou Medical University, Wenzhou, China

**Keywords:** Abnormal glucose metabolism, AIP index, CVD, CHARLS

## Abstract

**Background:**

The atherogenic index of plasma (AIP) and cardiovascular disease (CVD) in participants with abnormal glucose metabolism have been linked in previous studies. However, it was unclear whether AIP control level affects the further CVD incidence among with diabetes and pre-diabetes. Therefore, our study aimed to investigate the association between AIP control level with risk of CVD in individuals with abnormal glucose metabolism.

**Methods:**

Participants with abnormal glucose metabolism were included from the China Health and Retirement Longitudinal Study. CVD was defined as self-reporting heart disease and/or stroke. Using k-means clustering analysis, AIP control level, which was the log-transformed ratio of triglyceride to high-density lipoprotein cholesterol in molar concentration, was divided into five classes. The association between AIP control level and incident CVD among individuals with abnormal glucose metabolism was investigated multivariable logistic regression analysis and application of restricted cubic spline analysis.

**Results:**

398 (14.97%) of 2,659 participants eventually progressed to CVD within 3 years. After adjusting for various confounding factors, comparing to class 1 with the best control of the AIP, the OR for class 2 with good control was 1.31 (95% CI, 0.90–1.90), the OR for class 3 with moderate control was 1.38 (95% CI, 0.99–1.93), the OR for class 4 with worse control was 1.46 (95% CI, 1.01–2.10), and the OR for class 5 with consistently high levels was 1.56 (95% CI, 1.03–2.37). In restricted cubic spline regression, the relationship between cumulative AIP index and CVD is linear. Further subgroup analysis demonstrated that the similar results were observed in the individuals with agricultural Hukou, history of smoking, diastolic blood pressure ≥ 80mmHg, and normal body mass index. In addition, there was no interaction between the AIP control level and the subgroup variables.

**Conclusions:**

In middle-aged and elderly participants with abnormal glucose metabolism, constant higher AIP with worst control may have a higher incidence of CVD. Monitoring long-term AIP change will contribute to early identification of high risk of CVD among individuals with abnormal glucose metabolism.

**Supplementary Information:**

The online version contains supplementary material available at 10.1186/s12933-024-02144-y.

## Introduction

Cardiovascular diseases (CVDs) are the leading cause of death and disability globally [1, 2]. The overall prevalence of CVD has almost doubled with 271 million cases in 1990 to 523 million cases in 2019, and the number of deaths from CVD has steadily increased from 12.1 million in 1990 to 18.6 million in 2019 [2, 3]. Despite significant progress in drug treatment and interventional therapy in recent years, the number of deaths from CVD remains the highest, demonstrating a serious threat to public health. Thus, early identification of individuals with high risk of CVD will contribute to preventing disease progression.

Atherogenic Index of Plasma (AIP) is a new identified and lipid metabolism associated biomarker panel of plasma atherogenicity, which is calculated by the log-transformed ratio of Triglyceride (TG) to high-density lipoprotein cholesterol (HDL-C) in molar concentrations [4]. AIP was correlated with lipoprotein particle size and fractional esterification rate of HDL cholesterol (FERHDL) [5], which was closely related to insulin resistance [6, 7]. One study with 17,382 adult participants from the National Health and Nutrition Examination Survey (NHANES) had demonstrated high AIP was associated with 1.17-fold and 1.26-fold increased risk of all-cause mortality and CVD-specific mortality, respectively [8]. Subsequently another Kailuan study that collected 54,440 participants after 11.05 years of follow-up showed that participants with the highest levels of AIP had a significantly increased risk of myocardial infarction [9]. Alifu et al. studied an 8-fold increased risk of major adverse cardiovascular events in participants with chronic coronary syndromes compared to those with low AIP, suggesting that AIP also has significant prognostic predictive power in patients with CVD [10]. Most of these studies have focused on researching the relationship between AIP levels as a predictor of the incidence of various cardiovascular diseases (e.g., myocardial infarction and coronary heart disease) and the prognosis of patients with cardiovascular disease, suggesting that AIP is a strong predictive biomarker of the incidence of cardiovascular disease or the prognosis of patients with related diseases [8–10].

Indeed, AIP has been demonstrated to be a significant predictor of incident pre-diabetes or diabetes [11]. Glucose metabolism abnormality is prevalent in individuals with established CVD. Compared with normal glucose metabolism, individuals with type 2 diabetes mellitus (T2DM) and pre-diabetes are also significantly associated with poor outcomes among CVD individuals [12, 13]. Therefore, early identification of modifiable cardiovascular risk factors among individuals with abnormal glucose metabolism (a collective term for diabetes mellitus and pre-diabetes mellitus) will contribute to prevention of cardiovascular complications and early death [14, 15]. Given its prominent role in the development of pre-diabetes or diabetes, several studies further found the AIP was also an excellent predictor for risk of CVD among participants with abnormal glucose metabolism [16, 17]. However, it is unclear whether the level of AIP control influences further CVD incidence in participants with diabetes and prediabetes. Accordingly, our study aim to investigate the association between AIP control level with risk of CVD in individuals with abnormal glucose metabolism by using data from the China Health and Retirement Longitudinal Study (CHARLS).

## Methods

### Study design and population

Our study is based on data from CHARLS, which is a nationally representative longitudinal survey of persons aged 45 years in China. Supported by the multistage probability sampling method, the CHARLS baseline survey was conducted between 2011 and 2012 (wave 1). To date, CHARLS has made public three waves of follow-up data (wave 2 in 2013, wave 3 in 2015 and wave 4 in 2018) [18].

**Fig. 1:**
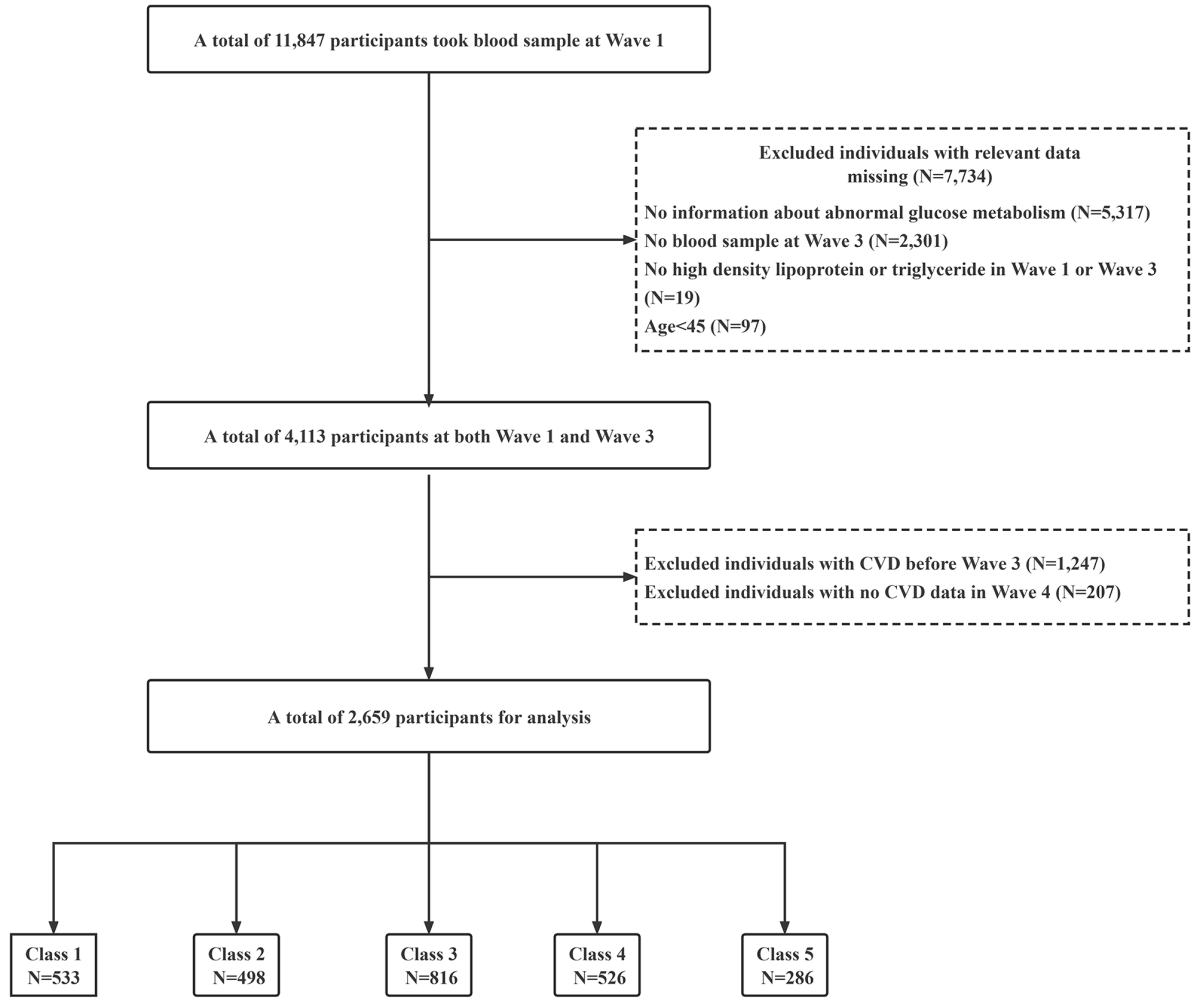
Flowchart of the study population

In this study, 11,847 participants who underwent a complete blood count test were included in the analysis. We excluded 8,484 participants for reasons including: (1) lack of diagnostic markers for abnormal glucose metabolism; (2) No complete blood cell test was performed on the wave 3; (3) Lack of TG or HDL-C level measurement; (4) under 45 years of age; (5) People who self-reported having CVD events before 2015. A total of 6,821 participants were finally included (Fig. 1).

CHARLS datasets are available for download at the CHARLS home website (http://charls.pku.edu.cn/en). The Biomedical Ethics Review Board of Peking University gave its ethical approval for the gathering of CHARLS data (IRB00001052-11015), and all participants signed an informed consent form.

### Determination of participants with abnormal glucose metabolism

Abnormal glucose metabolism including diabetes and prediabetes. According to American Diabetes Association (ADA) diabetes diagnostic criteria [19], prediabetes is defined as fasting plasma glucose (FPG) at 100–125 mg/dl or glycosylated hemoglobin A1c (HbA1c) at 5.7–6.4%; Diabetes with FPG ≥ 125 mg/dl, HbA1c ≥ 6.5%, self-reported medical history, or use of antidiabetic medications [20].

### Determination of CVD and calculation of AIP

CVD was the primary outcome event of the study, and heart disease and stroke were secondary outcome events. The presence of heart disease is determined by the question “Did your doctor tell you that you have been diagnosed with a heart attack, angina pectoris, coronary heart disease, heart failure, or other heart problem?” Similarly, the occurrence of stroke is ascertained through the question “Did your doctor tell you that you were diagnosed with a stroke?”. CVD is defined as self-reporting heart disease and/or stroke.

This study investigated AIP changes as exposures in Wave1 and Wave3 participants using k-means cluster analysis in a logistic regression equation where AIP was calculated as lg (TG/HDL-C). In addition, blood measurements from 2011 to 2012 were covered in Wave1, and measurements from 2015 were published in Wave3. Therefore, our cumulative AIP was determined by the expression: (AIP2012 + AIP2015)/2* time (2015 − 2012) [9].

### Ascertainment of covariates

Based on previous studies, we included potential confounding covariates at baseline. Demographic covariates included age, gender (“Male” and “Female”), education level (“Primary school or lower” and “Secondary school or higher”), current married (“Current marital” and “Others”), Hukou (government household registration system) (“Agriculture” and “Others”). Health behavior covariates included smoking status (“Yes”, “No”), drinking status (“Yes”, “No”), systolic blood pressure (SBP), diastolic blood pressure (DBP) and body mass index (BMI). Laboratory examination included fasting blood glucose (FBG), TG, blood urea nitrogen (BUN), total-cholesterol (TC), and HDL-C, Low-Density Lipoprotein Cholesterol (LDL-C), C-reactive protein (CRP), HbA1c and Uric acid (UA). There are some data missing in the CHARLS database, and the missing number and proportion of each variable are shown in Table S1. In order to ensure the accuracy of the research results, Template method (R Package “VIM”) [21]and multiple interpolation method (R Package “mice”) [22]were used to fill them.

### Statistical analysis

**Fig. 2:**
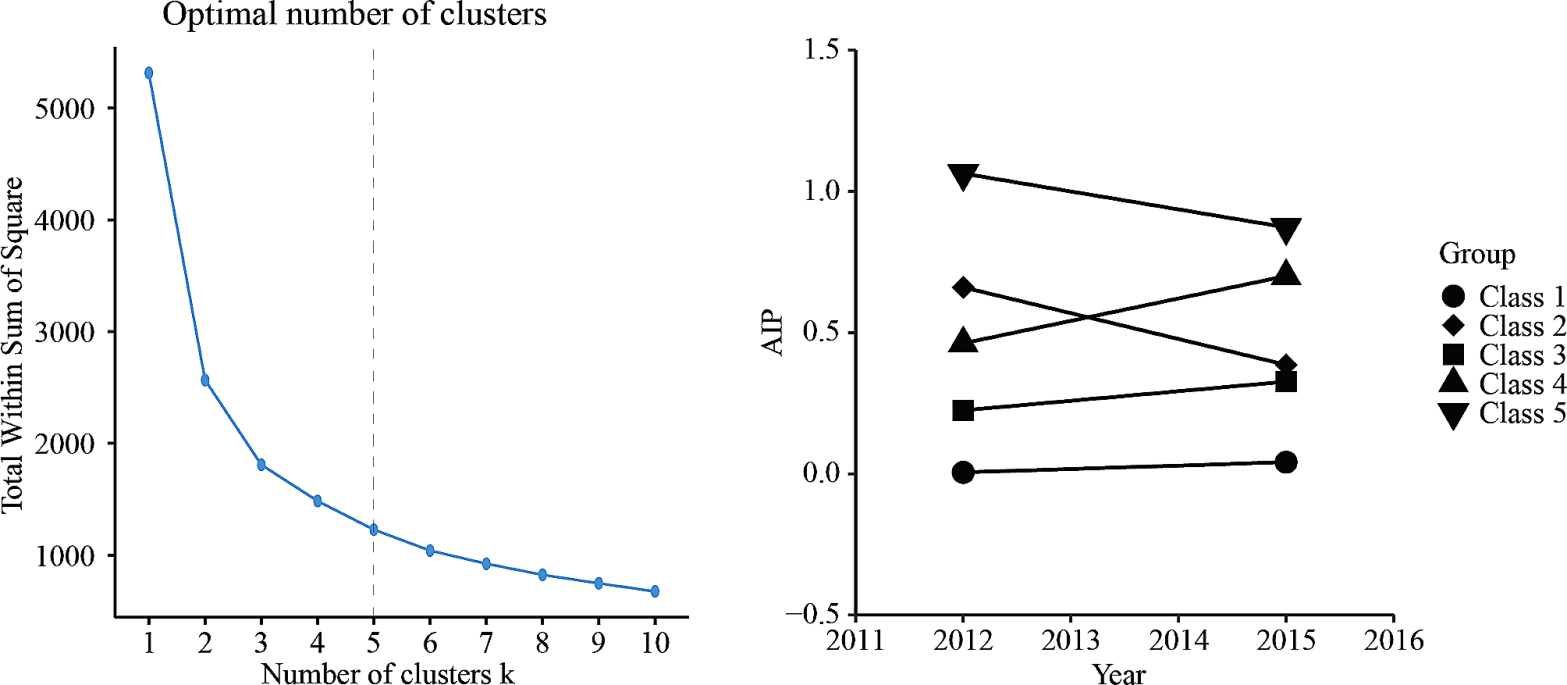
**(a)** K-means clustering method for clustering the atherosclerosis index of plasma; **(b)** The AIP clustering by k-means clustering

K-means clustering is a rule-based method for determining the distance between data items(Fig. 2A). The advantages of this approach are simplicity and scalability. In our study, when the number of clusters is equal to 5. K-means clustering works best [22, 23]: For class 1, AIP ranged from 0.016 to 0.037, representing the best AIP level control; For class 2, AIP ranged from 0.665 to 0.366, representing the better AIP level control; For class 3, AIP ranged from 0.231 to 0.325, representing an increase in AIP levels from a low level to a high level with poor control; For class 4, AIP ranged from 0.467 to 0.694, representing an increase in AIP levels from lower levels to higher levels, with worse control; For class 5, AIP ranged from 1.063 to 0.860, representing the worst AIP level of control (Fig. 2B).

The mean and standard deviation (SD) of continuous data, and percentage of classified data are used to describe basic features. Continuous variables were compared by Kruskal-Wallis rank sum test, and categorical variables were compared by Chi-square test or Fisher exact probability test. Hereafter, we used multivariable logistic regression analysis to establish three models to examine the relationship between AIP and CVD. The crude model was not adjusted for any variables. In model I, the data was adjusted for age, gender. And in model II, the results were adjusted for age, gender, education, marital status, residence location, smoking status, drinking status, SBP, DBP and BMI. The results from the logistic regression analysis are presented as odds ratios (ORs) and 95% confidence intervals (CIs). In addition, restricted cubic splines can reflect nonlinearity between variables. So, we used the restricted cubic spline model to test the correlation between cumulative AIP and CVD, and chose four knots at 20th, 40th, 60th and 80th. We further stratified analyses according to current married, age (Q1, Q2, Q3), gender, education level (primary school or lower, secondary school or higher), Hukou (agriculture, others), SBP (Q1, Q2, Q3), DBP (Q1, Q2, Q3), BMI (Q1, Q2, Q3), drinking status, current smoking status to examine whether potential confounding variables modulated the potential association between changes in AIP and CVD, and to test their interactions. CVD was the primary outcome, and heart failure and stroke were the secondary outcomes. In addition, we explored the association between changes in AIP levels and two secondary outcomes of heart disease and stroke using the same methodology as the CVD outcome. In all calculations, a two-sided P-value < 0.05 was required for statistical significance. All analyses were performed in R version 4.1.0 and EmpowerStats version 4.1 (www.empowerstats.com; X&Y Solutions Inc.).

## Results

### Baseline characteristics of participants

In this study, 2.659 participants with abnormal glucose metabolism were included, with an average age of 58.34 ± 8.34 years, 47.09% males. The Cumulative AIP of 1.22 ± 0.90, 567 participants eventually developed CVD (398 participants had heart disease and 169 had stroke) after a mean follow-up of 3 years. Compared to Class 1, participants in the other classes had a lower prevalence of current smoking and drinking; higher levels of SBP, DBP, BMI, FBG, TG, TC, HbA1c and UA, but lower levels of BUN and HDL-C (Table 1). In addition, we also provided a baseline description of the missing variables before interpolation, which is similar to the above results (Table S2).

**Table 1:** Baseline characteristics of participants with plasma atherogenic index measured

Characteristics	Total	Class 1	Class 2	Class 3	Class 4	Class 5	P-value
N	2659	533	498	816	526	286	
Age, years	58.34 ± 8.34	59.83 ± 8.39	57.22 ± 7.99	58.94 ± 8.60	57.79 ± 8.04	56.79 ± 8.06	< 0.001
Gender, n(%)							< 0.001
male	1252 (47.09%)	307 (57.60%)	222 (44.58%)	388 (47.55%)	216 (41.06%)	119 (41.61%)	
female	1407 (52.91%)	226 (42.40%)	276 (55.42%)	428 (52.45%)	310 (58.94%)	167 (58.39%)	
Education level, n(%)							0.038
Primary school or lower	1851 (69.61%)	379 (71.11%)	342 (68.67%)	589 (72.18%)	362 (68.82%)	179 (62.59%)	
Secondary school or higher	808 (30.39%)	154 (28.89%)	156 (31.33%)	227 (27.82%)	164 (31.18%)	107 (37.41%)	
Hukou, n(%)							0.005
Agriculture	2271 (85.41%)	482 (90.43%)	420 (84.34%)	694 (85.05%)	438 (83.27%)	237 (82.87%)	
Others	388 (14.59%)	51 (9.57%)	78 (15.66%)	122 (14.95%)	88 (16.73%)	49 (17.13%)	
Current married, n(%)	2400 (90.26%)	467 (87.62%)	452 (90.76%)	734 (89.95%)	482 (91.63%)	265 (92.66%)	0.113
Drink, n(%)	936 (35.20%)	240 (45.03%)	175 (35.14%)	281 (34.44%)	151 (28.71%)	89 (31.12%)	< 0.001
Current smoking, n(%)	782 (29.41%)	185 (34.71%)	135 (27.11%)	243 (29.78%)	144 (27.38%)	75 (26.22%)	0.027
SBP, mmHg	129.50 ± 20.29	125.79 ± 20.34	130.69 ± 19.71	128.22 ± 19.89	132.11 ± 20.47	133.23 ± 20.64	< 0.001
DBP, mmHg	75.53 ± 11.46	72.45 ± 11.31	76.69 ± 11.57	74.64 ± 10.68	77.18 ± 11.59	78.78 ± 11.86	< 0.001
BMI, kg/m^2^	23.96 ± 9.91	21.63 ± 2.90	24.63 ± 3.41	23.72 ± 16.92	25.14 ± 3.93	25.68 ± 4.14	< 0.001
BUN, mg/dl	15.94 ± 4.49	16.53 ± 4.85	15.28 ± 4.11	16.07 ± 4.58	16.01 ± 4.54	15.44 ± 3.83	< 0.001
Fasting blood-glucose, mg/dl	121.23 ± 40.56	114.78 ± 27.84	124.01 ± 42.99	116.55 ± 35.65	120.36 ± 37.71	143.33 ± 61.43	< 0.001
Total Cholesterol, mg/dl	198.96 ± 39.88	193.09 ± 34.18	200.67 ± 41.07	196.45 ± 37.45	199.81 ± 37.17	212.55 ± 53.66	< 0.001
Triglycerides, mg/dl	149.54 ± 137.28	70.21 ± 24.63	197.63 ± 77.64	93.65 ± 27.04	136.29 ± 43.80	397.45 ± 267.86	< 0.001
HDL-C, mg/dl	50.43 ± 15.97	67.56 ± 16.07	41.51 ± 8.70	54.71 ± 11.49	45.54 ± 9.40	30.84 ± 7.50	< 0.001
LDL-C, mg/dl	118.48 ± 36.58	113.63 ± 29.22	118.34 ± 38.30	125.13 ± 33.25	127.59 ± 33.96	92.07 ± 44.93	< 0.001
CRP, mg/l	2.75 ± 7.89	2.42 ± 9.09	2.61 ± 8.95	2.76 ± 7.78	3.27 ± 7.28	2.67 ± 3.93	< 0.001
HbA1c, % (mmol/mol)	5.48 ± 0.99	5.37 ± 0.84	5.53 ± 0.94	5.40 ± 0.92	5.53 ± 1.07	5.70 ± 1.31	0.004
UA, mg/dl	4.44 ± 1.23	4.26 ± 1.19	4.51 ± 1.19	4.33 ± 1.20	4.54 ± 1.25	4.85 ± 1.32	< 0.001
AIP_2012_	0.40 ± 0.37	0.00 ± 0.18	0.66 ± 0.16	0.22 ± 0.14	0.46 ± 0.18	1.06 ± 0.29	< 0.001
AIP_2015_	0.41 ± 0.30	0.04 ± 0.13	0.39 ± 0.14	0.33 ± 0.12	0.70 ± 0.14	0.87 ± 0.19	< 0.001
Cumulative AIP	1.22 ± 0.90	0.07 ± 0.33	1.57 ± 0.34	0.83 ± 0.23	1.74 ± 0.34	2.90 ± 0.53	< 0.001
Heart disease, n(%)	255 (9.59%)	43 (8.07%)	46 (9.24%)	82 (10.05%)	53 (10.08%)	31 (10.84%)	0.671
Stroke, n(%)	169 (6.36%)	21 (3.94%)	30 (6.02%)	52 (6.37%)	41 (7.79%)	25 (8.74%)	0.042
CVD, n(%)	398 (14.97%)	61 (11.44%)	73 (14.66%)	127 (15.56%)	87 (16.54%)	50 (17.48%)	0.095

Abbreviations: SBP: systolic blood pressure; DBP: diastolic blood pressure; BMI: body mass index; BUN: blood urea nitrogen; HDL-C: high-density lipoprotein cholesterol; LDL-C: low-density lipoprotein cholesterol; CRP: C-reactive protein; HbA1c: hemoglobin A1C; UA: uric acid; AIP: atherogenic index of plasma; CVD: cardiovascular disease

Notes: Continuous variables were expressed as mean ± standard deviation (SD) in case of normal distribution and compared between two groups by Kruskal-Wallis rank sum test. If the count variable had a theoretical number < 10, Fisher’s exact probability test was used. Categorical variables are presented as counts (percentages) and compared by Chi-square test

### Odds ratios for incident CVD

The logistic regression analyses are presented in Table 2 for the association between different classes of AIP and CVD. We construct three covariate models with different adjustments to reflect this relationship. After adjusting for various confounding factors, comparing to class 1, the ORs (95% CIs) for incident CVD were 1.31 (0.90, 1.90) for Class 2, 1.38 (0.99, 1.93) for Class 3, 1.46 (1.01, 2.10) for Class 4, and 1.56 (1.03, 2.37) for Class 5. Meanwhile class 4 and class 5 show that AIP has a significant correlation with high CVD incidence. In our secondary outcomes analysis, class 2–5 exhibited a significantly elevated risk of stroke compared to class 1. For heart disease, although the P-value did not reach significance, there was a progressive increase in risk observed for all classes except class 4(Table S3, Table S4).

**Table 2:** Logistic regression analysis for the association between different classes and CVD

Cluster	Case	Crude	Model I	Model II
		OR(95%CI) P-value	OR(95%CI) P-value	OR(95%CI) P-value
Total	398 (14.97%)	-	-	-
Class1	61 (11.44%)	Ref	Ref	Ref
Class2	73 (14.66%)	1.33 (0.92, 1.91) 0.126	1.39 (0.96, 2.00) 0.083	1.31 (0.90, 1.90) 0.159
Class3	127 (15.56%)	1.43 (1.03, 1.98) 0.033	1.42 (1.02, 1.97) 0.037	1.38 (0.99, 1.93) 0.057
Class4	87 (16.54%)	1.53 (1.08, 2.18) 0.017	1.56 (1.09, 2.23) 0.015	1.46 (1.01, 2.10) 0.042
Class5	50 (17.48%)	1.64 (1.09, 2.46) 0.017	1.72 (1.14, 2.59) 0.010	1.56 (1.03, 2.37) 0.037

Model I, adjusted for age, gender.

Model II, adjusted for age, gender, education, marital status, Hukou, smoking status, drinking status, systolic blood pressure, diastolic blood pressure and body mass index.

**Fig. 3:**
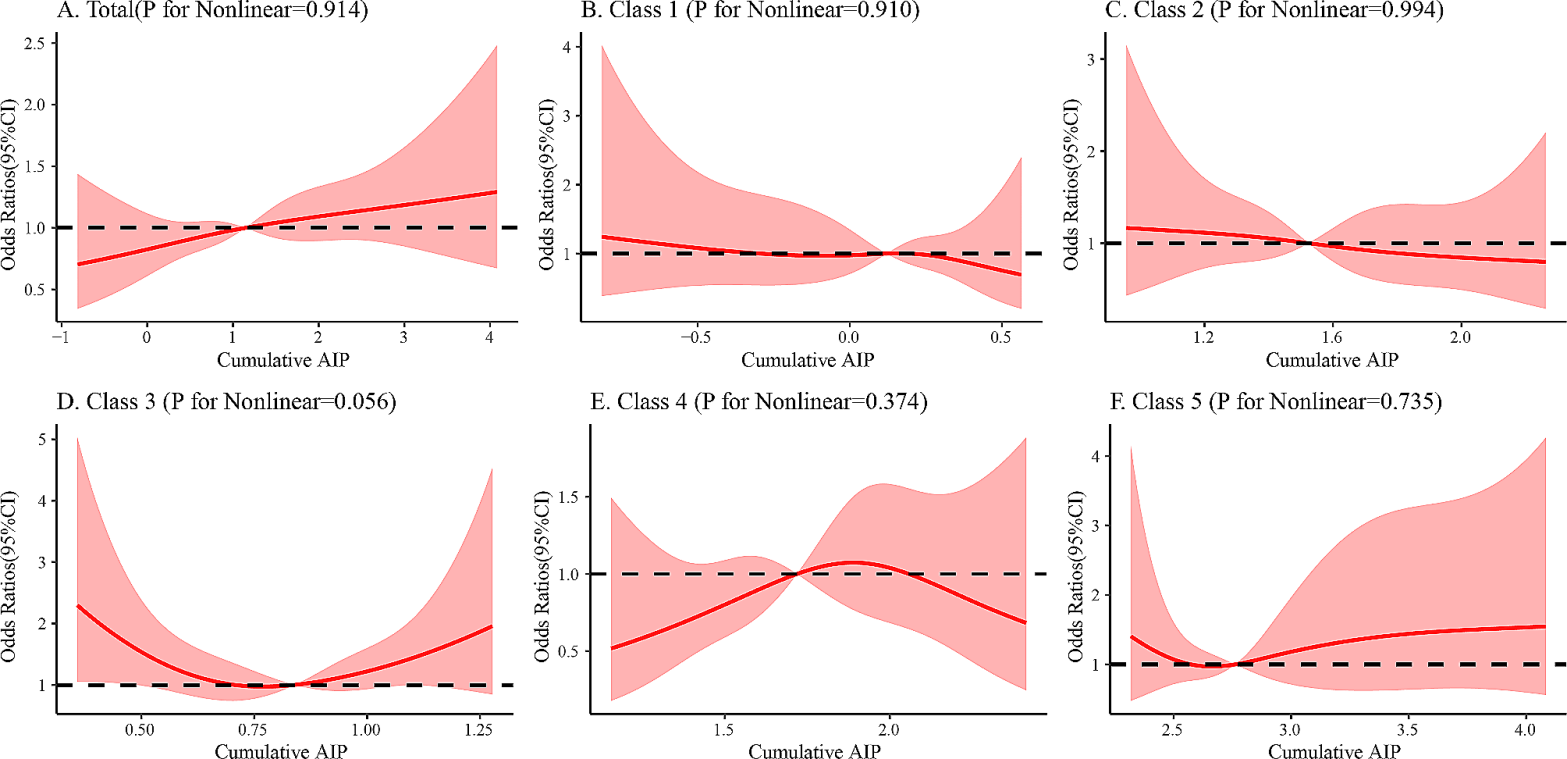
Cubic model of the association between different classes and cumulative AIP index after adjusting for age, gender, education, marital status, residence location, smoking status, drinking status, systolic blood pressure, diastolic blood pressure and body mass index

In the restricted cubic spline regression models shown in Fig. 3, the cumulative AIP and CVD risk are linear. The risk of CVD was increasing with each increase in the cumulative AIP above 1.12 (OR 1, 95% CI, 0.98, 1.01). However, in class 1 and class 2, the risk of CVD decreasing with each increase in the cumulative AIP; The values of class 4 OR were all less than 1, and both class 3 and class 5 were greater than 1.

### Subgroup analyses

We performed a subgroup analysis to explore the association of the AIP control level with incident CVD events stratified by potential risk factors. As shown in Table S5, subgroup analysis based on class1, class3-5 showed a higher risk of CVD in agricultural residence location (53 < Age < 61 years) and smoking patients compared to other subgroups.

No interaction was found between AIP control level and subgroup variables. For the two secondary outcomes of heart disease and stroke, the subgroup analysis was similar to that of CVD. It is worth noting that there is interaction between the two potential variables of gender and household registration in the stroke event (Table S6, Table S7).

## Discussion

To our knowledge, this is the first study to revealed a linear association between AIP control level with future stroke in middle-aged and elderly Chinese people with abnormal glucose metabolism, which demonstrated the worst control of AIP was a strong predictor of incident CVD among individuals with diabetes or pre-diabetes.

It is believed that there is a substantial correlation between AIP and LDL particle size [7]. It has been shown that the primary underlying cause of cardiovascular disorders including acute coronary syndrome and stroke is atherosclerosis [23]. And the onset and progression of atherosclerosis are determined by the quantity and size of LDL particles [24]. This is consistent with our secondary outcomes AIP and stroke findings that the risk of stroke increases with increasing AIP clustering. Second, AIP was also significantly associated with FERHDL [7], Fractional esterification rate of HDL cholesterol (FERHDL) was defined as the percentage of HDL free cholesterol (HDLFC) after depletion of apolipoprotein b during HDL development [5]. and a clinical trial confirmed an association between FERHDL and cardiovascular disease risk factors [25]. Another study showed that FERHDL and LDL particle size were predictors of insulin resistance [26, 27]. It is widely recognized that insulin resistance is a significant risk factor for cardiovascular disease in addition to being a prevalent underlying cause of diabetes [28, 29]. Therefore, it was shown that among people with diabetes or pre-diabetes, controlling AIP was a reliable predictor of incident CVD.

The results of this study showed that in the long-term follow-up, the AIP of class 2 was higher than that of class 3 and class 4 in the early stage, but that as time went on, it showed a declining trend and a commensurate decline in the risk of CVD. This implies that AIP is an indicator that can be controlled. Even if participants had high baseline AIP levels, when AIP levels were reduced after some intervention, the risk of later CVD also decreased. AIP was lowered by 57.85% and the incidence of hypercholesterolemia was dramatically decreased when the diabetic rats in the rat experiment were given lipid-lowering and hypoglycemic therapy [30]. Given that individuals with diabetes and prediabetes have greater AIP levels, these investigations provide additional support for our findings. It is even more necessary to control AIP levels to reduce the risk of cardiovascular disease. Therefore, AIP is considered to be an indicator with a strong association with diabetes and CVD, which can predict the risk of developing CVD with diabetes or pre-diabetes. However, further high-quality prospective trials are needed to confirm our findings.

We then discovered that the correlation between cumulative AIP and CVD produced some intriguing findings. The risk of CVD increases with increasing AIP level, despite the long-established linear association between AIP and CVD [31]. According to our research, people who have abnormal glucose metabolism may have a higher tolerance for cumulative AIP. There are also studies that show that when an individual’s AIP < 0.11, the risk of CVD is low, while the risk between 0.11 and 0.21 is medium, > 0.21 is high risk [31]. Interestingly, the risk of CVD is different for people with different levels of control. In the results of this study, for people with good AIP control level such as class1 and 2, the OR value decreased with the increase of cumulative AIP. However, for the poor AIP control level of class3-5, the OR values tended to increase, and the risk of CVD development varied significantly with the level of AIP control.

Subgroup analysis revealed that based on class1, class3-5 showed a higher risk of CVD in agricultural residence location (53 < Age < 61 years) and smoking patients compared to other subgroups. Previous evidence has long been clear that smoking, alcohol consumption and BMI are associated with the risk of CVD [32]. According to epidemiological studies, regular exercise protects against cardiovascular disease and type 2 diabetes, as well as the risk of death [33]. For example, 12 weeks of aerobic Nordic walking can reduce AIP levels in middle-aged men with impaired blood sugar regulation, thereby reducing the risk of developing diabetes and complications [34]. By modifying one’s diet, for as by eating enough peanuts, also can lower their risk of developing AIP and coronary heart disease [35].

Our study has various advantages. First, there are few studies on the relationship between AIP and new-onset CVD in people with abnormal glucose metabolism, and we are the first study to use cluster analysis to classify changes in AIP. The population was divided into well-controlled and poorly controlled groups based on the level of AIP change, and it was found that reducing AIP significantly reduced the incidence of cardiovascular events. Second, we used data from large-scale national longitudinal surveys and adjusted for multiple confounders, which can reflect the inner association between AIP control level and new-onset CVD in participants with chronic glucose metabolism abnormalities. Third, our study suggests that AIP, as an indicator of low cost and convenience, may have clinical implications for the treatment of new-onset CVD in patients with abnormal glucose metabolism. Moreover, these biochemical parameters can be conveniently obtained from a single sample at the same time, potentially improving the long-term prognosis of participants with abnormal glucose metabolism.

Our study also has limitations. First, due to the limited sample size, the sampling error is large, which may affect the accuracy and stability of the relationship between AIP and new-onset CVD in people with abnormal glucose metabolism. Second, after the exclusion of TG and HDL-C measurement individuals, it will lead to the loss of diabetic metabolic population, which may have an impact on the results. Third, the participants in this study were exclusively from China. While the findings may have broader implications, confirmation through similar studies is needed for global clinical guidelines. Fourth, the diagnosis of CVD is self-reported physician’s diagnosis, lacking further CVD event adjudication was performed in those individuals who responded that they had had a CVD event. Fifth, there was still 207 patients without CVD data in the 2018 excluded in our final analysis. Fortunately, the proportion of lost to follow-up is statistically acceptable.

## Conclusion

In this investigation, we discovered that while adequate control of AIP greatly reduces the risk of CVD, individuals with abnormal glucose metabolism may be more susceptible to CVD due to high baseline AIP and persistently higher AIP with poor control. Consequently, in daily practice, AIP should be regarded as a simple indicator of CVD, attention should be focused on preventing CVD in cases of abnormal glucose metabolism associated with high AIP, and intervention measures like appropriate exercise and improved lifestyle choices should be implemented to control AIP levels.

### Electronic supplementary material

Below is the link to the electronic supplementary material.

Supplementary Material 1

## Data Availability

Data is provided within the manuscript or supplementary information files.

## Abbreviations

AIP: Atherogenic index of plasma
CVD: Cardiovascular disease
CVDs: Cardiovascular diseases
T2DM: Type 2 diabetes mellitus
TG: Triglyceride
HDL-C: High-density lipoprotein cholesterol
NHANES: National Health and Nutrition Examination Survey
PER_HDL_: Particle size and fractional esterification rate of HDL-C
CHARLS: China Health and Retirement Longitudinal Study
ADA: American Diabetes Association
FPG: Fasting blood glucose
HbA1c: Hemoglobin A1c
SBP: Systolic blood pressure
DBP: Diastolic blood pressure
BMI: Body mass index
BUN: Blood urea nitrogen
LDL-C: Low-Density Lipoprotein Cholesterol
CRP: C-reactive protein
UA: Uric acid
SD: Standard deviation
