# Supplementary material for: Association between atherogenic index of plasma control level and incident cardiovascular disease in middle-aged and elderly Chinese individuals with abnormal glucose metabolism
Source: Cardiovasc Diabetol. 2024 Feb 8;23:54. doi: 10.1186/s12933-024-02144-y (PMC10854096; doi:10.1186/s12933-024-02144-y)
Supplement: Supplementary file 1 — Supplementary Material 1 [file 12933_2024_2144_MOESM1_ESM.docx]

**Table S1 Missing number for variables.**

| **Variables** | **Missing number (%)** |
| --- | --- |
| Age | 0 (0) |
| Gender | 0 (0) |
| Education level | 0 (0) |
| Current married | 0 (0) |
| Hukou | 16 (0.60%) |
| Smoking | 33 (1.24%) |
| Drinking | 1 (0.38%) |
| SBP | 310 (11.66%) |
| DBP | 309 (11.62%) |
| BMI | 311 (11.70%) |
| FBG | 55 (2.07%) |
| TG | 0 (0) |
| BUN | 0 (0) |
| TC | 0 (0) |
| HDL-C | 0 (0) |
| LDL-C | 9 (0.34%) |
| CRP | 0 (0) |
| HbA1c | 15 (0.56%) |
| UA | 0 (0) |

Abbreviations: SBP: systolic blood pressure; DBP: diastolic blood pressure; BMI: body mass index; FBG: fasting blood-glucose; TG: Triglycerides; BUN: blood urea nitrogen; TC: Total Cholesterol; HDL-C: high-density lipoprotein cholesterol; LDL-C: low-density lipoprotein cholesterol; CRP: C-reactive protein; HbA1c: [hemoglobin A1C](http://www.dictall.com/indu/214/213618925C9.htm); UA: uric acid.

**Table S2 Baseline Characteristics of missing variables before multiple interpolation.**

| **Characteristics** | **Total** | **Class 1** | **Class 2** | **Class 3** | **Class 4** | **Class 5** | P-value |
| --- | --- | --- | --- | --- | --- | --- | --- |
| N | 2659 | 533 | 498 | 816 | 526 | 286 |  |
| Current married, n (%) | 2400 (90.26%) | 467 (87.62%) | 452 (90.76%) | 734 (89.95%) | 482 (91.63%) | 265 (92.66%) | 0.113 |
| Hukou, n(%) |  |  |  |  |  |  | 0.006 |
| Agriculture | 2256 (85.36%) | 479 (90.38%) | 415 (84.35%) | 691 (84.99%) | 434 (83.14%) | 237 (82.87%) |  |
| Others | 387 (14.64%) | 51 (9.62%) | 77 (15.65%) | 122 (15.01%) | 88 (16.86%) | 49 (17.13%) |  |
| Smoking, n (%) | 773 (29.44%) | 185 (35.17%) | 133 (27.20%) | 239 (29.69%) | 142 (27.20%) | 74 (26.06%) | 0.015 |
| Drinking, n (%) | 936 (35.21%) | 240 (45.03%) | 175 (35.14%) | 281 (34.48%) | 151 (28.71%) | 89 (31.12%) | <0.001 |
| SBP, mmHg | 129.80 ± 20.33 | 126.12 ± 20.60 | 130.54 ± 19.50 | 128.73 ± 19.85 | 132.21 ± 20.42 | 134.11 ± 21.18 | <0.001 |
| DBP, mmHg | 75.61 ± 11.48 | 72.57 ± 11.45 | 76.39 ± 11.50 | 74.97 ± 10.67 | 77.15 ± 11.59 | 78.96 ± 12.10 | <0.001 |
| BMI, kg/m^2^ | 24.02 ± 10.46 | 21.58 ± 2.91 | 24.61 ± 3.38 | 23.90 ± 17.93 | 25.18 ± 3.96 | 25.77 ± 4.10 | <0.001 |
| FBG, mg/dl | 120.62 ± 39.62 | 114.63 ± 27.87 | 123.72 ± 43.06 | 116.06 ± 34.29 | 120.34 ± 37.86 | 140.71 ± 59.17 | <0.001 |
| LDL-C, mg/dl | 118.70 ± 36.21 | 113.63 ± 29.22 | 118.34 ± 38.30 | 125.13 ± 33.25 | 127.59 ± 33.96 | 93.29 ± 43.22 | <0.001 |
| HbA1c, % (mmol/mol) | 5.48 ± 1.00 | 5.37 ± 0.85 | 5.53 ± 0.94 | 5.40 ± 0.93 | 5.53 ± 1.07 | 5.70 ± 1.31 | <0.001 |

Abbreviations: SBP:systolic blood pressure; DBP: diastolic blood pressure; BMI: body mass index; FBG: fasting blood-glucose; LDL-C: low-density lipoprotein cholesterol; HbA1c: [hemoglobin A1C](http://www.dictall.com/indu/214/213618925C9.htm).

Notes: Continuous variables were expressed as mean±standard deviation (SD) in case of normal distribution and compared between two groups by Kruskal-Wallis rank sum test. If the count variable had a theoretical number <10, Fisher's exact probability test was used. Categorical variables are presented as counts (percentages) and compared by Chi-square test.

**Table S3 Logistic regression analysis for the association** **between different classes and heart disease.**

| **Cluster** | **Case** | **Crude** | **Model** **Ⅰ** | **Model** **Ⅱ** |
| --- | --- | --- | --- | --- |
|  |  | OR(95%CI) P-value | OR(95%CI) P-value | OR(95%CI) P-value |
| Total | 255 (9.59%) | - | - | - |
| Class1 | 43 (8.07%) | Ref | Ref | Ref |
| Class2 | 46 (9.24%) | 1.16 (0.75, 1.79) 0.504 | 1.14 (0.74, 1.78) 0.550 | 1.13 (0.72, 1.76) 0.593 |
| Class3 | 82 (10.05%) | 1.27 (0.87, 1.87) 0.221 | 1.22 (0.83, 1.80) 0.313 | 1.21 (0.81, 1.79) 0.351 |
| Class4 | 53 (10.08%) | 1.28 (0.84, 1.95) 0.256 | 1.22 (0.80, 1.87) 0.358 | 1.20 (0.78, 1.85) 0.410 |
| Class5 | 31 (10.84%) | 1.39 (0.85, 2.25) 0.189 | 1.35 (0.83, 2.21) 0.229 | 1.31 (0.79, 2.16) 0.291 |
| Model Ⅰ, adjusted for age, gender.  Model Ⅱ, adjusted for age, gender, education, marital status, Hukou, smoking status, drinking status, systolic blood pressure, diastolic blood pressure and body mass index. | | | | |

**Table S4 Logistic regression analysis for the association between different classes and stroke.**

| **Cluster** | **Case** | **Crude** | **Model Ⅰ** | **Model Ⅱ** |
| --- | --- | --- | --- | --- |
|  |  | OR (95%CI) P-value | OR (95%CI) P-value | OR (95%CI) P-value |
| Total | 169 (6.36%) | - | - | - |
| Class1 | 21 (3.94%) | Ref | Ref | Ref |
| Class2 | 30 (6.02%) | 1.56 (0.88, 2.77) 0.1257 | 1.78 (1.00, 3.17) 0.0512 | 1.61 (0.90, 2.89) 0.1109 |
| Class3 | 52 (6.37%) | 1.66 (0.99, 2.79) 0.0557 | 1.75 (1.04, 2.94) 0.0365 | 1.69 (0.99, 2.86) 0.0523 |
| Class4 | 41 (7.79%) | 2.06 (1.20, 3.54) 0.0087 | 2.31 (1.33, 3.99) 0.0028 | 2.07 (1.18, 3.61) 0.0107 |
| Class5 | 25 (8.74%) | 2.34 (1.28, 4.25) 0.0055 | 2.72 (1.48, 5.01) 0.0012 | 2.36 (1.27, 4.39) 0.0066 |
| Model Ⅰ, adjusted for age, gender.  Model Ⅱ, adjusted for age, gender, education, marital status, Hukou, smoking status, drinking status, systolic blood pressure, diastolic blood pressure and body mass index. | | | | |

**Table S5 Subgroup analysis of the associations between different classes and CVD.**

|  | **Case** | **Class 1** | **Class 2** | **Class 3** | **Class 4** | **Class 5** | ***P* for interaction** |
| --- | --- | --- | --- | --- | --- | --- | --- |
| Age, years |  |  |  |  |  |  | 0.204 |
| ≤53 | 782 | Ref | 1.32 (0.60, 2.90) 0.497 | 0.97 (0.44, 2.13) 0.942 | 0.91 (0.39, 2.15) 0.830 | 1.37 (0.56, 3.33) 0.487 |  |
| 53-61 | 893 | Ref | 2.12 (0.93, 4.86) 0.075 | 3.15 (1.49, 6.68) 0.003 | 3.21 (1.46, 7.10) 0.004 | 2.56 (1.03, 6.41) 0.044 |  |
| ≥61 | 984 | Ref | 1.01 (0.59, 1.73) 0.982 | 1.04 (0.66, 1.63) 0.866 | 1.14 (0.69, 1.90) 0.605 | 1.19 (0.64, 2.21) 0.576 |  |
| Gender |  |  |  |  |  |  | 0.425 |
| Male | 1252 | Ref | 1.48 (0.84, 2.60) 0.171 | 1.39 (0.85, 2.27) 0.191 | 2.04 (1.19, 3.49) 0.009 | 1.93 (1.00, 3.73) 0.050 |  |
| Female | 1407 | Ref | 1.14 (0.68, 1.91) 0.621 | 1.31 (0.82, 2.08) 0.262 | 1.10 (0.66, 1.83) 0.713 | 1.33 (0.75, 2.34) 0.325 |  |
| Education level |  |  |  |  |  |  | 0.874 |
| Primary school or lower | 1851 | Ref | 1.31 (0.84, 2.05) 0.228 | 1.48 (1.00, 2.19) 0.050 | 1.43 (0.92, 2.20) 0.110 | 1.68 (1.01, 2.79) 0.045 |  |
| Secondary school or higher | 808 | Ref | 1.17 (0.57, 2.39) 0.673 | 1.12 (0.58, 2.15) 0.744 | 1.35 (0.67, 2.71) 0.396 | 1.15 (0.52, 2.54) 0.739 |  |
| Current married |  |  |  |  |  |  | 0.452 |
| No | 259 | Ref | 1.67 (0.51, 5.52) 0.397 | 0.99 (0.34, 2.86) 0.988 | 2.02 (0.62, 6.55) 0.243 | 3.29 (0.83, 13.08) 0.091 |  |
| Yes | 2400 | Ref | 1.22 (0.82, 1.83) 0.326 | 1.39 (0.97, 1.98) 0.071 | 1.35 (0.91, 2.00) 0.141 | 1.40 (0.89, 2.20) 0.150 |  |
| Hukou |  |  |  |  |  |  | 0.727 |
| Agriculture | 2271 | Ref | 1.36 (0.91, 2.04) 0.131 | 1.44 (1.01, 2.06) 0.0453 | 1.58 (1.07, 2.34) 0.022 | 1.83 (1.17, 2.86) 0.008 |  |
| Others | 388 | Ref | 1.15 (0.39, 3.40) 0.804 | 1.36 (0.52, 3.58) 0.531 | 1.16 (0.40, 3.38) 0.779 | 0.76 (0.19, 3.01) 0.693 |  |
| Current smoking status |  |  |  |  |  |  | 0.267 |
| No | 1877 | Ref | 1.02 (0.66, 1.58) 0.921 | 1.12 (0.76, 1.65) 0.559 | 1.14 (0.75, 1.75) 0.539 | 1.14 (0.70, 1.86) 0.602 |  |
| Yes | 782 | Ref | 2.22 (1.01, 4.90) 0.047 | 2.30 (1.15, 4.60) 0.019 | 2.51 (1.16, 5.45) 0.020 | 3.30 (1.34, 8.17) 0.010 |  |
| Drink status |  |  |  |  |  |  | 0.111 |
| No | 1723 | Ref | 0.91 (0.57, 1.46) 0.686 | 1.08 (0.72, 1.64) 0.701 | 1.03 (0.65, 1.61) 0.904 | 1.24 (0.75, 2.07) 0.401 |  |
| Yes | 936 | Ref | 2.31 (1.22, 4.35) 0.010 | 1.98 (1.11, 3.54) 0.020 | 2.67 (1.41, 5.08) 0.003 | 1.97 (0.89, 4.35) 0.092 |  |
| SBP, mmHg |  |  |  |  |  |  | 0.677 |
| ≤118.5 | 864 | Ref | 0.89 (0.42, 1.86) 0.749 | 1.11 (0.60, 2.03) 0.743 | 1.15 (0.56, 2.38) 0.700 | 0.95 (0.39, 2.30) 0.908 |  |
| 118.5-136 | 884 | Ref | 1.85 (0.93, 3.70) 0.081 | 2.11 (1.16, 3.84) 0.014 | 1.56 (0.78, 3.12) 0.205 | 1.59 (0.70, 3.62) 0.271 |  |
| ≥136 | 911 | Ref | 1.22 (0.67, 2.20) 0.517 | 1.18 (0.68, 2.06) 0.556 | 1.45 (0.82, 2.58) 0.206 | 1.67 (0.89, 3.14) 0.113 |  |
| DBP, mmHg |  |  |  |  |  |  | 0.850 |
| ≤70 | 851 | Ref | 0.97 (0.49, 1.94) 0.942 | 1.08 (0.62, 1.90) 0.785 | 1.01 (0.51, 2.02) 0.969 | 1.30 (0.58, 2.96) 0.524 |  |
| 70-80 | 902 | Ref | 1.35 (0.71, 2.56) 0.356 | 1.38 (0.78, 2.41) 0.266 | 1.30 (0.68, 2.49) 0.425 | 1.09 (0.49, 2.42) 0.837 |  |
| ≥80 | 906 | Ref | 1.62 (0.81, 3.24) 0.174 | 1.90 (0.99, 3.65) 0.055 | 2.12 (1.09, 4.13) 0.028 | 2.25 (1.10, 4.60) 0.027 |  |
| BMI, kg/m^2^ |  |  |  |  |  |  | 0.617 |
| ≤21.99 | 886 | Ref | 1.34 (0.73, 2.48) 0.344 | 1.15 (0.72, 1.83) 0.561 | 1.21 (0.65, 2.26) 0.554 | 0.50 (0.14, 1.72) 0.269 |  |
| 21.99-24.90 | 885 | Ref | 1.43 (0.69, 2.96) 0.330 | 1.79 (0.94, 3.40) 0.076 | 1.62 (0.78, 3.35) 0.197 | 2.38 (1.09, 5.19) 0.029 |  |
| ≥24.90 | 888 | Ref | 1.29 (0.55, 2.99) 0.560 | 1.48 (0.63, 3.45) 0.365 | 1.52 (0.67, 3.45) 0.322 | 1.64 (0.69, 3.89) 0.259 |  |

Abbreviations: SBP: systolic blood pressure; DBP: diastolic blood pressure; BMI: body mass index.

Notes: In addition to the stratification variables themselves, age, gender, education, marital status, Hukou, smoking status, drinking status, SBP, DBP and BMI were adjusted.

**Table S6 Subgroup analysis of the associations between different classes and heart.**

|  | **Case** | **Class 1** | **Class 2** | **Class 3** | **Class 4** | **Class 5** | ***P* for interaction** |
| --- | --- | --- | --- | --- | --- | --- | --- |
| Age, years |  |  |  |  |  |  | 0.349 |
| ≤53 | 782 | Ref | 1.15 (0.45, 2.94) 0.768 | 0.70 (0.27, 1.83) 0.470 | 0.93 (0.34, 2.53) 0.889 | 1.10 (0.38, 3.20) 0.864 |  |
| 53-61 | 893 | Ref | 2.01 (0.78, 5.23) 0.150 | 2.81 (1.19, 6.62) 0.018 | 2.69 (1.07, 6.72) 0.035 | 1.82 (0.61, 5.41) 0.281 |  |
| ≥61 | 984 | Ref | 0.84 (0.44, 1.62) 0.606 | 0.96 (0.56, 1.64) 0.882 | 0.88 (0.48, 1.65) 0.700 | 1.16 (0.56, 2.38) 0.687 |  |
| Gender |  |  |  |  |  |  | 0.986 |
| Male | 1252 | Ref | 1.30 (0.64, 2.63) 0.462 | 1.24 (0.68, 2.28) 0.479 | 1.32 (0.65, 2.69) 0.438 | 1.49 (0.63, 3.51) 0.361 |  |
| Female | 1407 | Ref | 1.02 (0.57, 1.85) 0.942 | 1.16 (0.69, 1.98) 0.573 | 1.11 (0.63, 1.98) 0.710 | 1.23 (0.64, 2.35) 0.529 |  |
| Education level |  |  |  |  |  |  | 0.884 |
| Primary school or lower | 1851 | Ref | 1.00 (0.59, 1.71) 0.997 | 1.18 (0.75, 1.86) 0.477 | 1.23 (0.74, 2.03) 0.432 | 1.33 (0.73, 2.43) 0.353 |  |
| Secondary school or higher | 808 | Ref | 1.48 (0.63, 3.47) 0.371 | 1.36 (0.62, 2.98) 0.447 | 1.15 (0.48, 2.75) 0.758 | 1.30 (0.50, 3.42) 0.588 |  |
| Current married |  |  |  |  |  |  | 0.614 |
| No | 259 | Ref | 2.92 (0.63, 13.59) 0.172 | 2.04 (0.52, 7.99) 0.304 | 1.10 (0.17, 7.18) 0.919 | 3.22 (0.45, 22.81) 0.242 |  |
| Yes | 2400 | Ref | 1.03 (0.64, 1.65) 0.916 | 1.14 (0.75, 1.73) 0.541 | 1.17 (0.73, 1.85) 0.516 | 1.22 (0.71, 2.07) 0.471 |  |
| Hukou |  |  |  |  |  |  | 0.784 |
| Agriculture | 2271 | Ref | 1.06 (0.65, 1.71) 0.822 | 1.16 (0.76, 1.77) 0.484 | 1.24 (0.78, 1.97) 0.362 | 1.36 (0.80, 2.32) 0.259 |  |
| Others | 388 | Ref | 2.16 (0.56, 8.26) 0.261 | 2.22 (0.64, 7.70) 0.207 | 1.54 (0.39, 6.13) 0.540 | 1.95 (0.41, 9.37) 0.404 |  |
| Current smoking status |  |  |  |  |  |  | 0.220 |
| No | 1877 | Ref | 0.86 (0.52, 1.44) 0.575 | 0.96 (0.62, 1.50) 0.859 | 0.99 (0.61, 1.63) 0.979 | 0.98 (0.55, 1.74) 0.951 |  |
| Yes | 782 | Ref | 2.61 (0.93, 7.26) 0.067 | 2.56 (1.03, 6.34) 0.042 | 2.20 (0.77, 6.26) 0.1412 | 3.79 (1.15, 12.46) 0.028 |  |
| Drink status |  |  |  |  |  |  | 0.232 |
| No | 1723 | Ref | 0.76 (0.44, 1.32) 0.329 | 0.92 (0.57, 1.47) 0.719 | 0.87 (0.52, 1.47) 0.601 | 0.96 (0.53, 1.76) 0.903 |  |
| Yes | 936 | Ref | 2.26 (1.02, 5.00) 0.045 | 1.96 (0.95, 4.05) 0.070 | 2.23 (0.97, 5.09) 0.058 | 2.11 (0.80, 5.55) 0.131 |  |
| SBP, mmHg |  |  |  |  |  |  | 0.471 |
| ≤118.5 | 864 | Ref | 0.68 (0.30, 1.55) 0.355 | 0.68 (0.34, 1.36) 0.277 | 0.99 (0.45, 2.16) 0.974 | 0.75 (0.28, 2.00) 0.572 |  |
| 118.5-136 | 884 | Ref | 1.95 (0.84, 4.52) 0.119 | 2.17 (1.04, 4.52) 0.039 | 1.89 (0.83, 4.32) 0.129 | 1.73 (0.65, 4.64) 0.274 |  |
| ≥136 | 911 | Ref | 1.06 (0.49, 2.26) 0.889 | 1.23 (0.62, 2.46) 0.551 | 0.88 (0.41, 1.91) 0.7521 | 1.38 (0.61, 3.10) 0.440 |  |
| DBP, mmHg |  |  |  |  |  |  | 0.622 |
| ≤70 | 851 | Ref | 1.10 (0.51, 2.40) 0.806 | 1.03 (0.54, 1.98) 0.921 | 0.77 (0.33, 1.78) 0.539 | 1.48 (0.60, 3.68) 0.394 |  |
| 70-80 | 902 | Ref | 1.36 (0.64, 2.89) 0.418 | 1.10 (0.55, 2.21) 0.779 | 1.31 (0.61, 2.81) 0.493 | 0.97 (0.37, 2.54) 0.943 |  |
| ≥80 | 906 | Ref | 0.75 (0.31, 1.78) 0.512 | 1.43 (0.67, 3.02) 0.352 | 1.27 (0.58, 2.81) 0.551 | 1.26 (0.53, 3.00) 0.609 |  |
| BMI, kg/m^2^ |  |  |  |  |  |  | 0.873 |
| ≤21.99 | 886 | Ref | 0.96 (0.44, 2.08) 0.914 | 0.98 (0.56, 1.71) 0.930 | 1.02 (0.47, 2.17) 0.969 | 0.71 (0.20, 2.53) 0.598 |  |
| 21.99-24.90 | 885 | Ref | 1.54 (0.67, 3.54) 0.311 | 1.47 (0.69, 3.13) 0.320 | 1.26 (0.52, 3.04) 0.612 | 2.23 (0.91, 5.48) 0.081 |  |
| ≥24.90 | 888 | Ref | 0.98 (0.37, 2.59) 0.961 | 1.35 (0.52, 3.52) 0.542 | 1.20 (0.47, 3.07) 0.708 | 1.12 (0.41, 3.07) 0.822 |  |

Abbreviations: SBP: systolic blood pressure; DBP: diastolic blood pressure; BMI: body mass index.

Notes: In addition to the stratification variables themselves, age, gender, education, marital status, Hukou, smoking status, drinking status, SBP, DBP and BMI were adjusted.

**Table S7 Subgroup analysis of the associations between different classes and stroke**

|  | **Case** | **Class 1** | **Class 2** | **Class 3** | **Class 4** | **Class 5** | **P for interaction** |
| --- | --- | --- | --- | --- | --- | --- | --- |
| Age, years |  |  |  |  |  |  | 0.417 |
| ≤53 | 782 | Ref | 1.99 (0.51, 7.80) 0.321 | 1.78 (0.46, 6.84) 0.402 | 0.91 (0.19, 4.36) 0.902 | 2.76 (0.66, 11.64) 0.166 |  |
| 53-61 | 893 | Ref | 2.03 (0.51, 8.07) 0.312 | 2.98 (0.84, 10.63) 0.092 | 3.87 (1.06, 14.14) 0.041 | 5.07 (1.25, 20.54) 0.023 |  |
| ≥61 | 984 | Ref | 1.33 (0.60, 2.95) 0.477 | 1.38 (0.71, 2.70) 0.347 | 1.99 (0.98, 4.06) 0.058 | 1.27 (0.50, 3.21) 0.614 |  |
| Gender |  |  |  |  |  |  | 0.464 |
| Male | 1252 | Ref | 1.53 (0.69, 3.39) 0.300 | 1.45 (0.72, 2.91) 0.296 | 2.71 (1.32, 5.57) 0.007 | 2.35 (0.97, 5.69) 0.058 |  |
| Female | 1407 | Ref | 1.69 (0.68, 4.23) 0.261 | 1.92 (0.82, 4.54) 0.135 | 1.59 (0.64, 3.95) 0.322 | 2.46 (0.95, 6.33) 0.063 |  |
| Education level |  |  |  |  |  |  | 0.457 |
| Primary school or lower | 1851 | Ref | 1.83 (0.91, 3.68) 0.092 | 2.09 (1.12, 3.93) 0.021 | 2.03 (1.02, 4.03) 0.043 | 2.75 (1.29, 5.87) 0.009 |  |
| Secondary school or higher | 808 | Ref | 1..06 (0.35, 3.24) 0.920 | 0.84 (0.30, 2.36) 0.736 | 1.73(0.63, 4.80) 0.290 | 1.26 (0.39, 4.08) 0.697 |  |
| Current married |  |  |  |  |  |  | 0.046 |
| No | 259 | Ref | 0.60 (0.11, 3.44) 0.571 | 0.35 (0.08, 1.56) 0.170 | 2.40 (0.65, 8.93) 0.191 | 2.44 (0.47, 12.72) 0.290 |  |
| Yes | 2400 | Ref | 1.82 (0.94, 3.53) 0.075 | 2.09 (1.14, 3.83) 0.016 | 2.03 (1.06, 3.88) 0.033 | 2.40 (1.18, 4.87) 0.016 |  |
| Hukou |  |  |  |  |  |  | 0.064 |
| Agriculture | 2271 | Ref | 1.79 (0.94, 3.41) 0.077 | 1.96 (1.10, 3.49) 0.023 | 2.43 (1.31, 4.48) 0.005 | 3.29 (1.70, 6.36) ＜0.001 |  |
| Others | 388 | Ref | 0.95 (0.21, 4.39) 0.948 | 0.83 (0.21, 3.20) 0.781 | 0.99 (0.23, 4.33) 0.990 | 0.00 (0.00, Inf) 0.991 |  |
| Current smoking status |  |  |  |  |  |  | 0.782 |
| No | 1877 | Ref | 1.30 (0.65, 2.60) 0.464 | 1.40 (0.75, 2.63) 0.292 | 1.51 (0.77, 2.97) 0.230 | 1.79 (0.85, 3.76) 0.125 |  |
| Yes | 782 | Ref | 2.14 (0.70, 6.48) 0.180 | 2.24 (0.82, 6.08) 0.114 | 3.38 (1.21, 9.45) 0.020 | 3.41 (1.04, 11.12) 0.042 |  |
| Drink status |  |  |  |  |  |  | 0.674 |
| No | 1723 | Ref | 1.38 (0.63, 3.05) 0.420 | 1.57 (0.77, 3.20) 0.213 | 1.57 (0.74, 3.35) 0.239 | 2.26 (1.01, 5.06) 0.046 |  |
| Yes | 936 | Ref | 1.94 (0.79, 4.73) 0.147 | 1.81 (0.81, 4.03) 0.149 | 3.06 (1.31, 7.13) 0.010 | 2.11 (0.71, 6.25) 0.176 |  |
| SBP, mmHg |  |  |  |  |  |  | 0.552 |
| ≤118.5 | 864 | Ref | 1.29 (0.30, 5.48) 0.734 | 2.87 (0.91, 9.09) 0.072 | 1.76 (0.43, 7.23) 0.4301 | 1.96 (0.40, 9.70) 0.408 |  |
| 118.5-136 | 884 | Ref | 1.54 (0.54, 4.34) 0.418 | 1.74 (0.72, 4.21) 0.219 | 1.42 (0.51, 3.96) 0.501 | 1.38 (0.40, 4.77) 0.607 |  |
| ≥136 | 911 | Ref | 1.67 (0.71, 3.90) 0.238 | 1.29 (0.56, 2.95) 0.552 | 2.38 (1.06, 5.33) 0.035 | 2.69 (1.13, 6.42) 0.025 |  |
| DBP, mmHg |  |  |  |  |  |  | 0.235 |
| ≤70 | 851 | Ref | 0.49 (0.13, 1.91) 0.307 | 1.28 (0.53, 3.10) 0.582 | 1.36 (0.48, 3.87) 0.560 | 0.90 (0.22, 3.69) 0.882 |  |
| 70-80 | 902 | Ref | 1.42 (0.52, 3.85) 0.493 | 1.65 (0.72, 3.81) 0.238 | 1.77 (0.67, 4.69) 0.248 | 1.55 (0.47, 5.06) 0.470 |  |
| ≥80 | 906 | Ref | 5.47 (1.55, 19.38) 0.008 | 3.83 (1.09, 13.49) 0.037 | 5.45 (1.56, 19.05) 0.008 | 7.57 (2.11, 27.15) 0.002 |  |
| BMI, kg/m^2^ |  |  |  |  |  |  | 0.326 |
| ≤21.99 | 886 | Ref | 2.20 (0.92, 5.26) 0.077 | 1.45 (0.70, 3.03) 0.320 | 1.71 (0.67, 4.35) 0.261 | 0.57 (0.07, 4.60) 0.600 |  |
| 21.99-24.90 | 885 | Ref | 0.93 (0.26, 3.35) 0.910 | 2.55 (0.93, 7.04) 0.070 | 2.37 (0.78, 7.24) 0.128 | 2.80 (0.82, 9.52) 0.100 |  |
| ≥24.90 | 888 | Ref | 1.60 (0.44, 5.81) 0.478 | 1.18 (0.31, 4.48) 0.807 | 1.80 (0.51, 6.38) 0.361 | 2.39 (0.66, 8.70) 0.185 |  |

Abbreviations: SBP: systolic blood pressure; DBP: diastolic blood pressure; BMI: body mass index.

Notes: In addition to the stratification variables themselves, age, gender, education, marital status, Hukou, smoking status, drinking status, SBP, DBP and BMI were adjusted.
